# Annexin A1 translocates to nucleus and promotes the expression of pro-inflammatory cytokines in a PKC-dependent manner after OGD/R

**DOI:** 10.1038/srep27028

**Published:** 2016-07-18

**Authors:** Baoming Zhao, Jing Wang, Lu Liu, Xing Li, Shuangxi Liu, Qian Xia, Jing Shi

**Affiliations:** 1 Department of Neurobiology, Tongji Medical College, Huazhong University of Science and Technology, 13 Hangkong Road, Wuhan 430030, P. R. China; 2 Key Laboratory of Neurological Diseases of Hubei Province, Tongji Medical College, Huazhong University of Science and Technology, 13 Hangkong Road, Wuhan 430030, P. R. China; 3 Institute for Brain Research, Huazhong University of Science and Technology, Wuhan 430030, Hubei Province, P. R. China; 4 Clinical laboratory, Center hospital of Wuhan, Wuhan 430030, Hubei Province, P. R. China

## Abstract

Annexin A1 (ANXA1) is a protein known to have multiple roles in the regulation of inflammatory responses. In this study, we find that after oxygen glucose deprivation/reoxygenation (ODG/R) injury, activated PKC phosphorylated ANXA1 at the serine 27 residue (p27S-ANXA1), and promoted the translocation of p27S-ANXA1 to the nucleus of BV-2 microglial cells. This in turn induced BV-2 microglial cells to produce large amounts of pro-inflammatory cytokines. The phenomenon could be mimicked by either transfecting a mutant form of ANXA1 with its serine 27 residue converted to aspartic acid, S27D, or by using the PKC agonist, phorbol 12-myristate 13-acetate (PMA) in these microglial cells. In contrast, transfecting cells with an ANXA1 S27A mutant (serine 27 converted to alanine) or treating the cells with the PKC antagonist, GF103209X (GF) reversed this effet. Our study demonstrates that ANXA1 can be phosphorylated by PKC and is subsequently translocated to the nucleus of BV-2 microglial cells after OGD/R, resulting in the induction of pro-inflammatory cytokines.

Annexin A1 (ANXA1), a member of the vertebrate annexin class A family of proteins, also previously known as lipocortin 1, has received more and more attention in light of recent research findings^1^,^2^. ANXA1 has been known to have multiple roles in important biological processes such as cell differentiation^3^, proliferation^4^, plasma membrane repair^5^, epithelial repair^6^, and cell apoptosis^7^. For instance, ANXA1 can bind to negatively charged cellular phospholipids, vesicles and cytoskeletal proteins such as F-actin^8^, demonstrating a possible role in intracellular trafficking^9^. In addition, there is compelling evidence of a role of extracellular ANXA1 in multiple anti-inflammatory processes^10^,^11^, including regulation of neutrophil migration, macrophage phagocytosis^12^,^13^, and induction of changes in cell polarity of microglial cells after ischemia like injury *in vitro*^14^,^15^. Although the function of ANXA1 in the central nervous system is still elusive, enhanced ANXA1 expression has been found at demyelinating brain lesions in patients suffering from multiple sclerosis^16^, Parkinson’s disease^17^, and Alzheimer’s disease^1^. Our recent data showed that ANXA1 can be translocated into the nucleus following oxygen glucose deprivation/reoxygenation (OGD/R injury) to induce neuronal cell death^7^, a role that is currently being examined in detail. Together these data highlight multiple roles of ANXA1 under normal and pathological conditions.

Protein kinase C (PKC) can be activated during ischemic injury in multiple tissues, including the heart^18^, liver^19^, and kidney^20^, which suggests a conserved role in the ischemic response pathway. However, whether PKC is a direct mediator of this pathway or is simply activated during ischemic-like injury remains controversial because of contradictory reports on the expression level, activity and distribution of PKC after injury. This is, therefore the main focus of our studies. There are also conflicting standpoints about the role of PKC in ischemic tissues, which are subject to debate. Some studies have shown that PKC levels and PKC activity increased after ischemia damage based on measurements taken at relatively early time points in some *in vivo* models^21^, as well as after OGD/R treatments, and excitotoxic injury *in vitro*^22^. Moreover, treating cells with PKC nonspecific inhibitors protected cells against excitotoxic cell death *in vitro*^23^,^24^ and against ischemic-like injury *in vivo*^25^. Another finding suggests that PKC acts as a switch to amplify pro-inflammatory pathways, which would be sufficient to induce neuronal cell death^26^. Taken together these findings indicate that PKC is activated during ischemia-like injury and may play a detrimental role in the pathophysiology of ischemic injury. Albeit, the precise molecular mechanisms underlying these biological processes are still not fully understood.

It was recently demonstrated that microglial cells were not only immunocentric, but in addition exerted neurobiological functions in both healthy and pathological contexts. In the disease context, the widespread consensus is that microglial cells are in a dynamic state with a potential to contribute to both central nervous system damage and repair^27^. The emerging roles of microglial cells are currently being investigated in the healthy and diseased brain with a growing interest in their diverse functions^27^. Interestingly, microglial cells are now being considered to be the CNS counterparts of peripheral macrophages, given the fact that they respond rapidly (within minutes) to immunological stimuli together with a burst of pro-inflammatory mediators^28^,^29^. Indeed, a number of studies have found that microenvironmental conditions can selectively modify unique microglia phenotypes and functions. The location of microglia in the ischemic brain changes their activation and cell fate. In the ischemic core, where blood flow is reduced to near zero, cell death is nearly universal by 24 hours^30^. Moreover constitutive expression of ANXA1 has been reported in glial cells scattered throughout normal adult human brains and in rodent, and specifically in microglial cells^9^,^31^. Thus, ANXA1, appears to be constitutively expressed in cells of the innate immune system of the normal brain, but whether it plays a role in regulating microglia function or not remains elusive.

In this study, we demonstrate an OGD/R related mechanism mediating the production of pro-inflammatory cytokines by BV-2 microglial cells. Following OGD/R injury, we found that increased PKC expression and activity leads to phosphorylation of ANXA1, which promotes its translocation to the nucleus, and induces the production of pro-inflammatory mediators by BV-2 microglial cells.

## Results

### OGD/R injury induce the pro-inflammatory action of BV-2 and primary microglial cells

**Figure 1:**
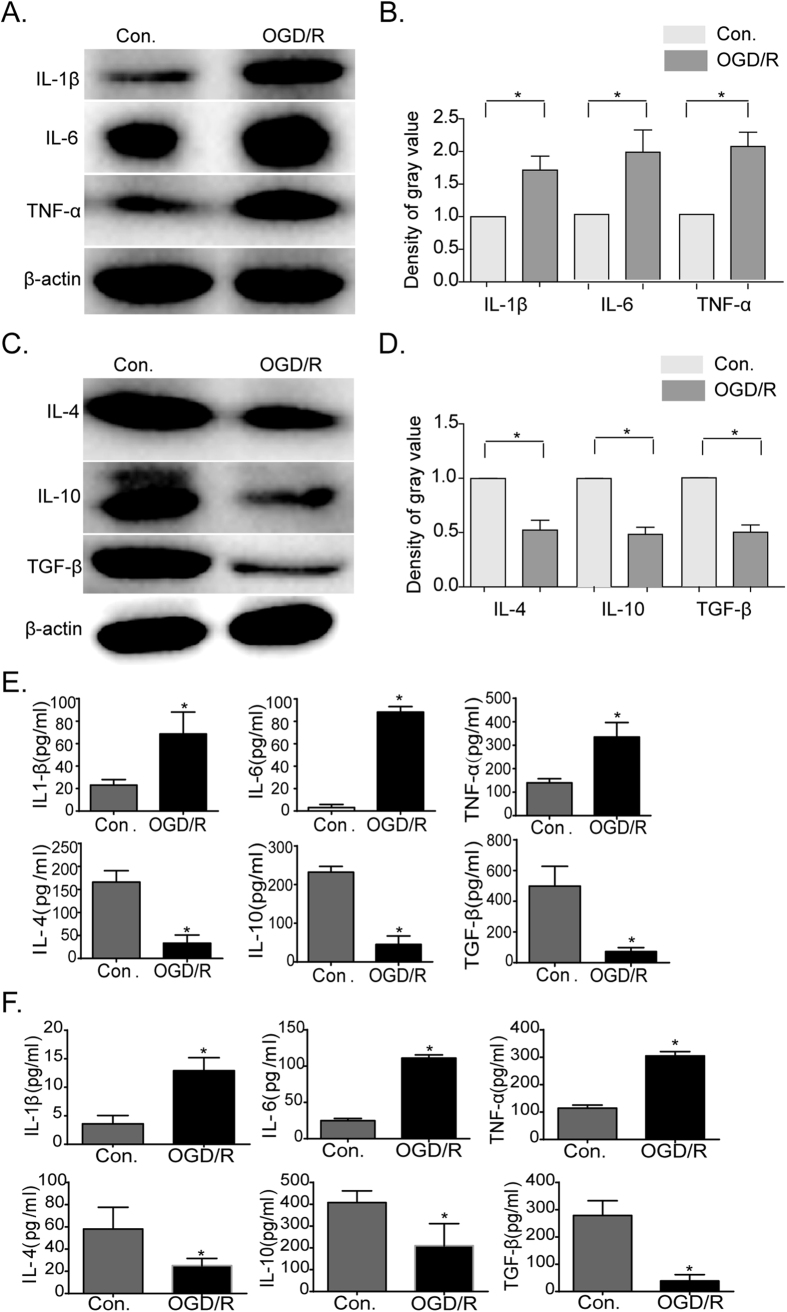
Expression and secretion of inflammatory cytokines in BV-2 and primary microglia cells under normal conditions and after OGD/R injury. (**A**) Western blot analysis of BV-2 microglial cell lysates showing the expression of IL-1β, IL-6, and TNF-α before and after OGD/R treatments. (**B**) Western blot quantifications of IL-1β, IL-6, and TNF-α intensities normalized to their respective controls (defined as 1.0). Data are expressed as mean ± SEM; n = 3; *P < 0.05 versus controls. (**C**) Western blots showing expression levels of IL-4, IL-10, and TGF-β in BV-2 microglia whole cell lysates. (**D**) Western blot quantifications of IL-4, IL-10, and TGF-β intensities normalized to their respective controls (defined as 1.0). Data are expressed as mean ± SEM; n = 3; *P < 0.05 versus controls. (**E**) ELISA detection of secreted lymphokines in supernatants of BV-2 microglial as well as (**F**) of isolated primary microglial cells. Data are expressed as mean ± SEM; n = 3; *P < 0.05 versus controls.

Thus far, the pivotal pathological progression of brain ischemia-reperfusion is post-ischemic inflammation, and the release of inflammatory cytokines from immune cells like microglial cells. In order to detect the levels of inflammatory cytokines released from BV-2 microglial cells under ischemia conditions, whole cell lysates and cell culture supernatants were collected from microglial cell cultures following OGD/R and subjected to SDS-PAGE analysis or, ELISA detection respectively. Our results show that the expression levels of the pro-inflammatory cytokines, IL-1β, IL-6 and TNF-α were higher when compared to those of non-hypoxic conditions (Fig. 1A,B, Fig. S2). In contrast, the levels of the anti-inflammatory cytokines, IL-4, IL-10, and TGF-β were found to be lower than the levels of in the non-hypoxic controls (Fig. 1C,D). Importantly, the levels of pro- and anti-cytokine secretion correlate with the cellular expression levels of the same cytokine in both BV-2 and primary microglial cells (Fig. 1E,F).

### ANXA1 is phosphorylated by PKC after OGD/R treatments

**Figure 2:**
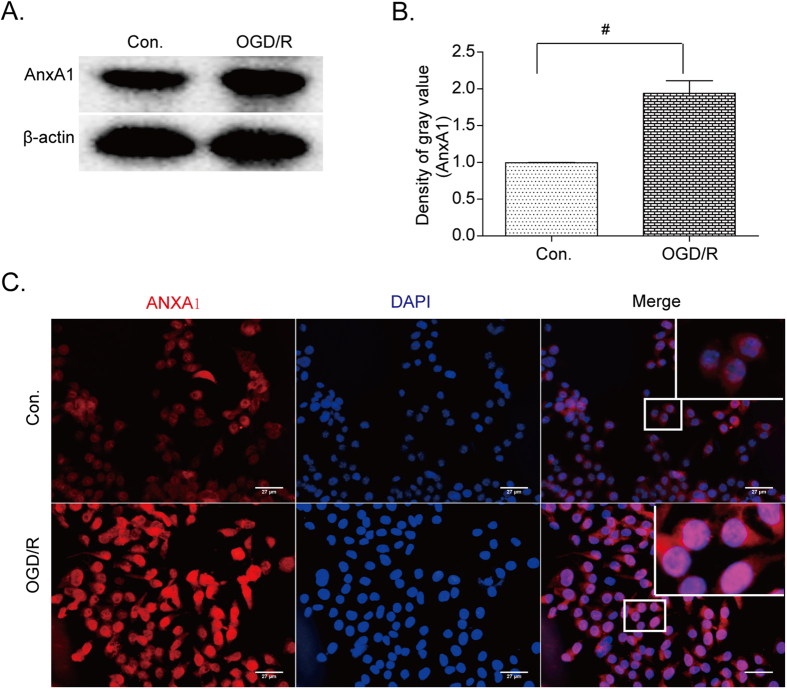
Expression and translocation of ANXA1 after OGD/R treatments. (**A**) Western blot analysis showing ANXA1 protein levels in BV-2 microglial cells before and after OGD/R. (**B**) Western blot intensities were quantified and normalized to their respective controls (defined as 1.0). Data are expressed as mean ± SEM; n = 3; ^#^*P* < 0.01 versus controls. (**C**) Immunofluorescence analysis of ANXA1 (red, left panels) in BV-2 microglial cells and in nuclei (blue, middle panels). Merged images are shown in the overlay pictures with partially enlarged details (right panels) in both control (top panels) and OGD/R conditions (bottom panels). Data are representative of three independent experiments. Bar = 27 μm.

ANXA1 has been considered a pivotal player in the pathological progression of the inflammatory response^2^. However, most previous studies have focused on the anti-inflammatory and pro-repair action of ANXA1, based on the expression and translocation of ANXA1 to the membrane and/or its secretion into the extracellular matrix (ECM)^1^,^32^. In this study, we explored the protein expression levels of ANXA1 in BV-2 microglial cells and found that ANXA1 protein levels were higher following OGD/R injury as detected by SDS-PAGE analysis (Fig. 2A,B), as well as by ANXA1 antibody staining of BV-2 microglial cells (Fig. 2C, compare top panels with bottom panels).

**Figure 3:**
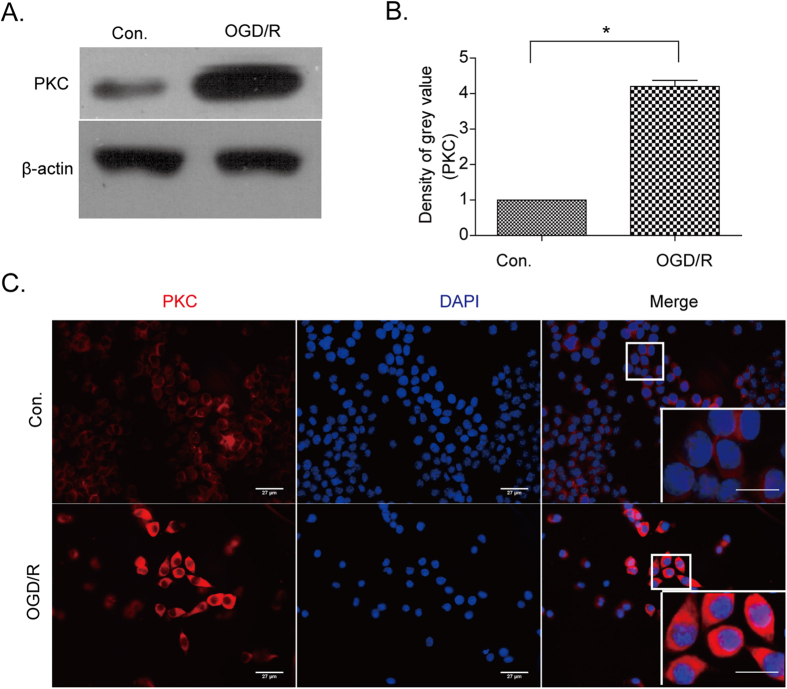
Expression of PKC in BV-2 microglial cells after OGD/R treatments. (**A**) Western blots showing protein levels of PKC in BV-2 microglial cell lysates under normal conditions and after OGD/R treatments. (**B**) Western blot intensities of PKC expression levels were quantified and normalized to their respective controls (defined as 1.0). Data are expressed as mean ± SEM; n = 3; *P < 0.01 versus control. (**C**) Immunocytochemistry analysis of PKC (red, left panels) in BV-2 microglial cells and their nuclei (blue, middle panels). Merged images are shown in the overlay pictures with partially enlarged details (right panels) in both control (top panels) and OGD/R conditions (bottom panels). Data are representative of three independent experiments. Bar = 27 μm.

**Figure 4:**
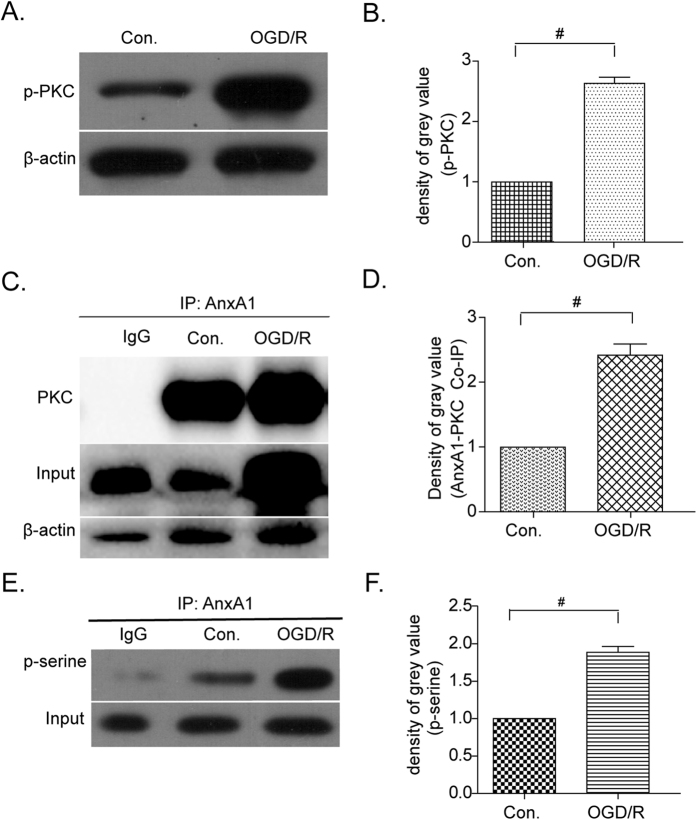
Phosphorylation levels of PKC and ANXA1 and their protein-protein interaction after OGD/R treatments. (**A**) PKC phosphorylation levels in BV-2 microglial cells after OGD/R treatment as detected with an anti-phospho-PKC polyclonal antibody. (**B**) Quantification of western blots intensities normalized to their respective controls (defined as 1.0). Data are expressed as mean ± SEM; n = 3; ^#^*P* < 0.01 versus controls. (**C**) Co-immunoprecipitation of PKC with ANXA1 in BV-2 microglial cells under negative (IgG), control (Con) and OGD/R conditions as indicated. **(D**) Quantification of western blot intensities normalized to their respective controls (defined as 1.0). Data are expressed as mean ± SEM; n = 3; ^#^*P* < 0.01 versus appropriate controls. (**E**) Serine phosphorylation levels of ANXA1 as detected with a specific phospho-serine polyclonal antibody after immunoprecipitation. (**F**) Quantification of western blot intensities normalized to their respective controls (defined as 1.0). Data are expressed as mean ± SEM; n = 3; ^#^*P* < 0.01 versus controls.

Furthermore, it has been demonstrated that PKC activation can have multiple effects on ischemia/reperfusion-mediated nervous system damage^23^,^33^. For instance, our previous data showed that in neurons, phosphorylation of ANXA1 at serine 5, mediated by transient receptor potential melastatin 7 (TRPM7) allows ANXA1 to be translocated to the nucleus to induce cell death after OGD/R injury^7^. In addition, Varticovski and colleagues reported that ANXA1 can be identified as a PKC substrate and in turn can be phosphorylated by PKC on the ANXA1 serine residue at position 27^34^. Consequently, we want to examine further whether PKC was activated by oxygen deprivation and whether the residue 27 of ANXA1 could indeed be phosphorylated by PKC. To this end, we explored the expression of PKC in BV-2 microglial cells using immunocytochemistry and immunofluorescence analysis. Our data showed that PKC protein levels were upregulated in whole cell lysates (Fig. 3A,B) and in immunofluorescence labeling of BV-2 cells following OGD/R injury (Fig. 3C, bottom panels). We also found that PKC activity was upregulated after OGD/R as detected by a pan phospho-PKC antibody (Fig. 4A,B).

Next, we performed co-immunoprecipitation (co-IP) studies to further investigate the relationship between PKC and ANXA1 after OGD/R injury. In our co-IP studies we found upregulated levels of total and active PKC proteins associated with ANXA1 under OGD/R conditions when compared to controls (Fig. 4C,D, compare lane 3 to lanes 1–2). Lastly, we used a serine phospho-specific poly-clonal antibody to detect the phosphorylation level of ANXA1 by PKC. In our SDS-PAGE analysis of BV-2 microglial cell lysates after OGD/R treatments, we found an increase in the phosphorylation levels of ANXA1 serine residue after OGD/R injury when compared to controls (Fig. 4E,F, compare lane 3 to lanes 1–2, Fig. S1).

### Translocation of phosphorylated ANXA1 to the nucleus of BV-2 cells

**Figure 5:**
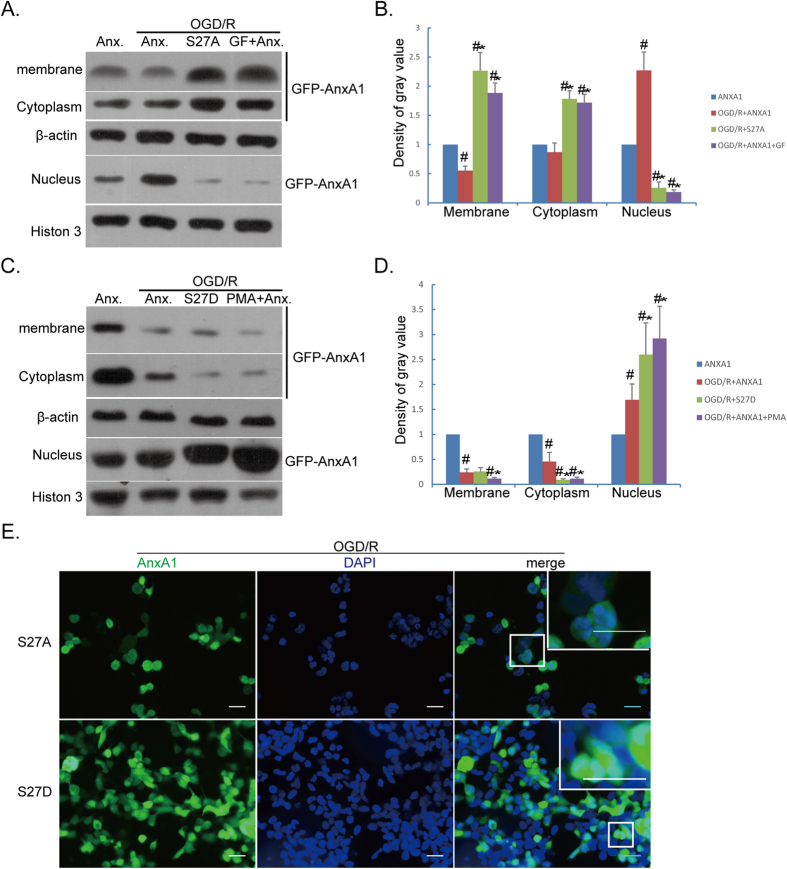
Translocation of ANXA1 after OGD/R injury. BV-2 microglial cells were transfected with wild-type (WT) ANXA1 or mutated ANXA1-S27A, or WT ANXA1 with addition of the PKC antagonist, GF109203X, (1 μM); or the ANXA1-S27D mutant or WT ANXA1 together with the PKC activator, phorbol ester (PMA, 1 μM). (**A**) Western blot analysis showing the translocation of ANXA1 in BV-2 microglial cells treated as indicated. The top western blot panel indicates the levels of translocated ANXA1 at the plasma membrane; in the cytoplasm (second panel from top), and in the nucleus (fourth panel from top). (**B**) Western blots intensities normalized to their respective controls (defined as 1.0). Data are expressed as mean ± SEM; n = 3; ^#^*P* < 0.05 versus ANXA1. ^#^*P < 0.05 versus ANXA1 + OGD/R. (**C**) Western blots showing the translocation of ANXA1 in BV-2 microglia cells treated as indicated. Top western blot panel indicates the levels of translocated ANXA1 at the plasma membrane; in the cytoplasm (second panel from top), and in nucleus (fourth panel from top). (**D**) Western blot intensities normalized to their respective controls (defined as 1.0). Data are expressed as mean ± SEM; n = 3; ^#^*P* < 0.01 versus ANXA1. ^#^*P < 0.05 versus ANXA1 + OGD/R. (**E**) Transfected EGFP-tagged ANXA1 constructs into BV-2 microglial cells (in green, left panels), and their nuclei (in blue, middle panels). Merged images are shown in the overlay pictures with partially enlarged details (right panels) for ANXA-S27A (top panels) and ANXA1-S27D (bottom panels). Bar = 20 μm. Data are representative of three independent experiments.

Together the data shown above demonstrate the presence of high protein levels of ANXA1 (Fig. 2C), upregulated levels of total PKC (Fig. 3A–C), active PKC (Fig. 4A,B), and PKC associated with ANXA1 (Fig. 4C,D), in BV-2 microglial cells after OGD/R treatments. We next sought to investigate whether PKC activation affected the translocation of ANXA1 to the nucleus. For this, we used EGFP-tagged constructs of ANXA1 that were subjected to site-directed mutagenesis at the serine 27 residue, the site shown to be phosphorylated by PKC^34^. In addition, we used an agonist and an antagonist of PKC to examine the effect of active PKC signaling on the distribution of the ANXA1 protein. Interestingly, the mutant protein ANXA1 S27A (with serine 27 mutated to alanine) was strongly translocated to the cell membrane after OGD/R treatments (Fig. 5A,B, compare lane 3 to lanes 1–2; and e, top panels), a phenomenon that could also be observed using the PKC antagonist, GF109203X, which resulted in decreased ANXA1 protein levels in the nucleus (Fig. 5A,B, compare lane 4 to lanes 1–2).

On the contrary, the mutant protein ANXA1-S27D (with serine 27 mutated to aspartic acid) into BV-2 microglial cells resulted in decreased ANXA1 protein levels in the plasma membrane and cytoplasm, and increased levels of translocated ANXA1 in the nucleus (Fig. 5C,D, compare lane 3 to lanes 1–2; and e, bottom panels), a similar effect on ANXA1 protein levels was seen after treatment of BV-2 microglial cells with phorbol ester (PMA) (Fig. 5C,D; compare lane 4 to lanes 1–2). We then wondered whether nuclear translocation of ANXA1 affected the pro-inflammatory action of BV-2 microglial cells.

### ANXA1 translocates to the nucleus to promote the pro-inflammatory action of BV-2 microglial cells after OGD/R treatments

**Figure 6:**
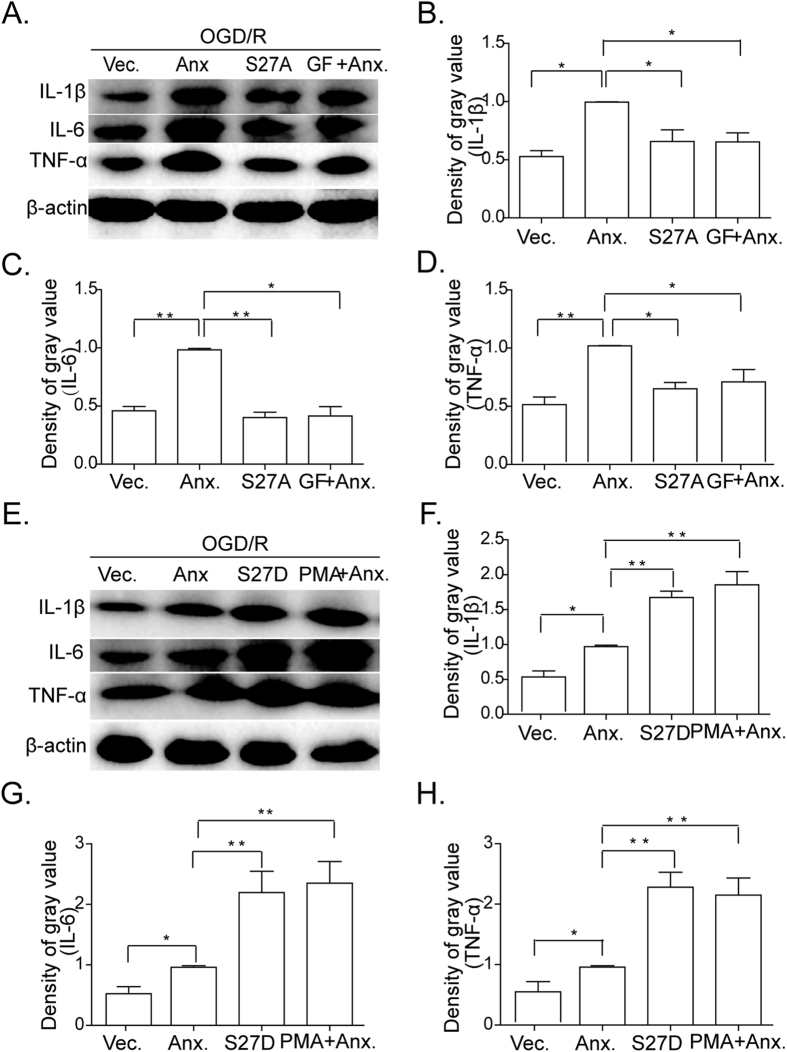
Translocation of ANXA1 affects the expression of pro-inflammatory cytokines in BV-2 microglial cells. Western blot analysis showing the expression of IL-1β, IL-6, and TNF-α in BV-2 microglia cells. (**A**) BV-2 microglial cells were transfected with either vector control, WT ANXA1, or ANXA1-S27A mutant, or WT ANXA1 together with GF109203X (1 μM) after OGD/R treatments. (**B–D**) Western blot intensities of IL-1β, IL-6, and TNF-α normalized to their respective controls (defined as 1.0). Data are presented as mean ± SEM for three independent experiments. Asterisks indicate statistically significant difference (*P < 0.05, **P < 0.01). (**E**) BV-2 microglial cells transfected with either vector, or WT ANXA1, or with ANXA1-S27D, or WT ANXA1 with addition of PMA (1 μM) after OGD/R treatments. (**F–H**) Western blot intensities of IL-1β, IL-6, and TNF-α normalized to their respective controls (defined as 1.0). Data are presented as mean ± SEM for three independent experiments. Asterisks indicate statistically significant difference (*P < 0.05, **P < 0.01).

Given that ANXA1 was previously thought to be a secreted protein and exert an anti-inflammatory and pro-repare action in ischemic tissue^1^, we asked whether ANXA1 could affect the expression of inflammatory cytokines in OGD/R induced inflammation. To do this, we set out to examine the relationship between the different subcellular distributions of ANXA1 and the upregulation of inflammatory cytokines. When BV-2 microglial cells were transfected with ANXA1-S27A constructs following by OGD/R treatment, the pro-inflammatory cytokines, IL-1β, IL-6, and TNF-α, were found to be expressed at lower levels than those of control groups (Fig. 6A–D, compare lane 3 to lanes 1–2). The same phenomenon could be seen using the PKC antagonist, GF109203X (Fig. 6A–D, compare lane 4 to lanes 1–2). In contrast, BV-2 microglial cells transfected with the ANXA1-S27D mutant (Fig. 6E–H, lane 3) or treated with PMA (Fig. 6E–H, lane 4) exhibited a reversed outcome with higher levels of pro-inflammatory cytokines expressed (Fig. 6E–H, compare lanes 3–4 with lanes 1–2, Fig. S3).

**Figure 7:**
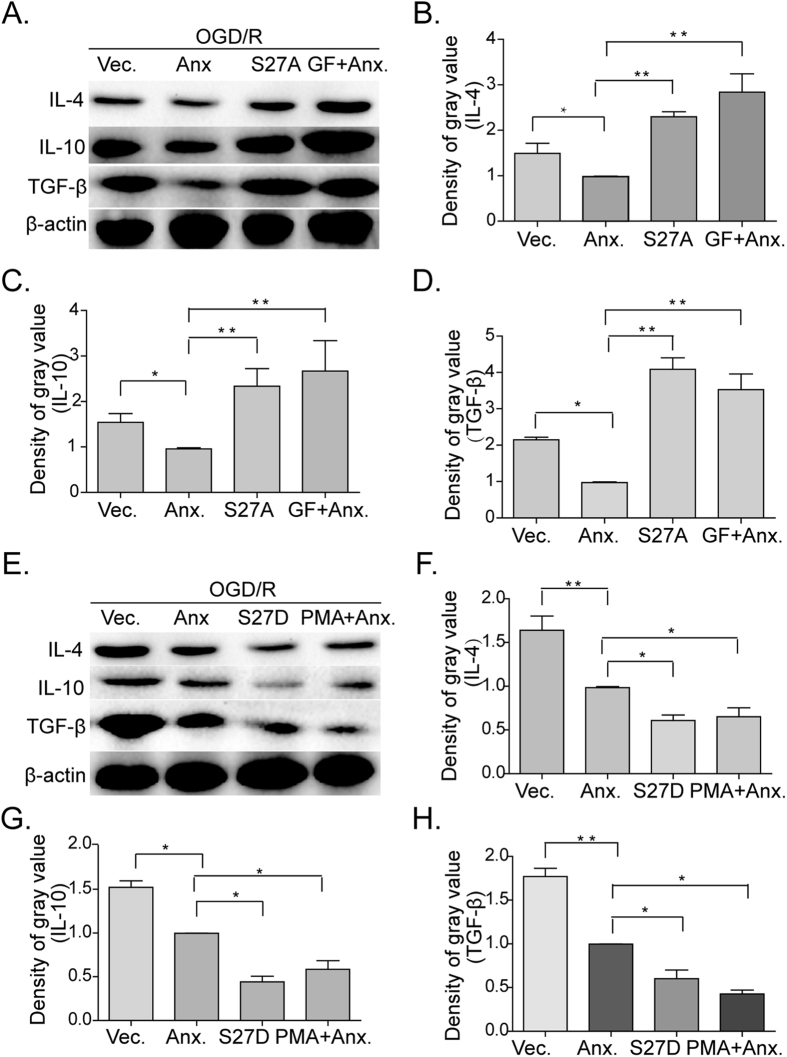
Translocation of ANXA1 to the nucleus affected the expression of anti-inflammatory cytokines in BV-2 microglial cells. Western blot showing the expression of IL-4, IL-10, and TGF-β in BV-2 microglia cells. (**A**) BV-2 microglial cells were transfected with either vector control alone, WT ANXA1, mutated ANXA1-S27A, or WT ANXA1 with addition of GF109203X (1 μM) and subjected to analysis after OGD/R treatments. (**B–D**) Western blot intensities of IL-4, IL-10, and TGF-β normalized to their respective controls (defined as 1.0). Data are presented as mean ± SEM for three independent experiments. Asterisks indicate statistically significant difference (*P < 0.05, **P < 0.01). (**E**) BV-2 microglial cells were transfected with either vector control alone, WT ANXA1, mutated ANXA1-S27D, or WT ANXA1 with addition of PMA (1 μM) and analyzed after OGD/R treatments. (**F–H**) Western blot intensities of IL-4, IL-10, and TGF-β were quantified and normalized to their respective controls (defined as 1.0). Data are presented as mean ± SEM for three independent experiments. Asterisks indicate statistically significant difference (*P < 0.05, **P < 0.01).

Next, we investigated the expression levels of the anti-inflammatory cytokines, IL-4, IL-10, and TGF-β, using the conditions described above and found that ANXA1-S27A and GF109203X treatments resulted in the increased expression of these anti-inflammatory cytokines (Fig. 7A–D). In contrast, when BV-2 microglial cells were transfected with ANXA1-S27D or treated with PMA, the anti-inflammatory cytokines examined here were expressed at much lower levels than in control groups (Fig. 7E–H).

## Discussion

In this study, we report an increase in PKC levels and activity after OGD/R treatment of BV-2 microglial cells. In addition, active PKC was found in association with ANXA1, which led to the phosphorylation of ANXA1 on serine 27. Phosphorylated ANXA1 was in turn translocated to the nucleus of BV-2 microglial cells, where it promoted the production of pro-inflammatory cytokines, while actively suppressing the production of anti-inflammatory cytokines after OGD/R treatment. These effects could be prevented using the PKC antagonist, GF109203X or by transfecting BV-2 microglial cells with an unphosphorylatable ANXA1 construct containing a serine to alanine mutation at position 27 (S27A). In contrast, using phorbol ester (PMA) to activate the PKC pathway, or a serine to aspartic acid mutant (S27D) of ANXA1 that renders it constitutively active, enhanced the production of pro-inflammatory cytokines in BV-2 microglial cells.

ANXA1 is an endogenous protein known to have potential anti-inflammatory functions in the peripheral nervous system^9^. However, ANXA1’s role in regulating inflammatory activities of the central nervous system (CNS) remains poorly understood. A few studies have proposed a protective role of ANXA1 in the CNS ischemic response^35^,^36^ as well as in the progression of neurodegenerative diseases^37^,^38^.

Previous studies have reported an anti-inflammatory and pro-resolving function of ANXA1 in the nervous system. In a recent publication, our group showed that enhanced ANXA1 binding to the formyl peptide receptor (FPR) induces morphological changes in microglial cells to an alternative phenotype of M2 polarized cells, which protects neurons against ischemia-like injury.

Using rodent microglia cultures, it was shown that the N-terminal fragment of ANXA1, Ac2–26, prevents lipopolysaccharide (LPS) mediated stimulation of cyclo-oxygenase 2 (COX-2) and inducible nitric oxide synthase (iNOS), as well as the release of nitric oxide (NO)^39^,^40^. In addition, ANXA1 is known to inhibit phospholipase A2 activity^41^, thereby preventing the release of arachidonic acid (AA), an essential fatty acid for prostanoid synthesis. Within the hypothalamic regulatory center, ANXA1 is also thought to mediate the antipyretic actions of glucocorticoid (GCs)^42^. These functions, in addition to the blocking of prostaglandin E2 (PGE2) synthesis, include the inhibition of the pro-inflammatory cytokines, IL-1β, IL-6 and IL-8, all of which are notably elevated in the striatum and cerebrospinal fluid (CSF) in subjects with idiopathic Parkinson’s disease^43^,^44^. Intra-cerebral administration of ANXA1 fragments also inhibits neuroendocrine and febrile responses to peripheral or centrally administered cytokines^45^,^46^. Furthermore, ANXA1 can bind to cell surface receptors to exert paracrine or autocrine effects on multiple biological events as described above. Although this so far eluded full confirmation, a growing body of evidence indicates that the effects of ANXA1 in the immune^47^ and neuroendocrine systems^9^,^48^ might be mediated by the FPR family of receptor proteins. The functional implications of ANXA1 gene expression changes in microglia and astrocytes are still unknown, but they can be used as potential targets for limiting neuro-inflammation and combatting neurodegeneration. Of note, these described actions of ANXA1 are mostly based on the translocation of ANXA1 to the plasma membrane and its subsequent secretion into the extracellular matrix.

Importantly, ANXA1 has been shown to have multiple opposing physiological roles. In our studies, we show that translocation of ANXA1 into neuronal nuclei after OGD/R injury, induces neuronal death. We have also found that ANXA1 is translocated to the nucleus of BV-2 microglial cells (Fig. 2C) and induces cells to produce pro-inflammatory cytokines (Fig. 6E–H, Fig. S3C,D), while it also suppresses the production of anti-inflammatory cytokines (Fig. 7E–H).

PKC has been implicated in mediating ischemia/reperfusion lesions in multiple organs^20^,^49^. Recent reports have linked PKC activity to regulatory events in several signaling pathways, including the mediation of excitatory or inhibitory amino acid release^50^, and cytokine induced superoxide production^51^. Here we found that the expression and activation of PKC was upregulated in BV-2 microglia cells after OGD/R treatments, which resulted in increased levels of ANXA1 that were co-immunoprecipitated with PKC. *In vivo* experiments of ^32^P-labeled mesangial cells, phosphorylation was increased by treating the cells with PKC activators, such as angiotensin II or by using common phorbol esters (i.e. PMA, TPA). Moreover, a phosphoamino acid analysis, revealed that phosphorylation of ANXA1 occurs only on serine residues^52^. Consistent with this we found that phosphorylation of ANXA1 at serine 27 residue in BV-2 microglial cells was upregulated after OGD/R treatment (Fig. 4E), implying a role for PKC in ANXA1 phosphorylation in microglial cells after OGD/R injury.

Importantly, most studies elucidating the role of PKC pathways, report a rapid loss of total PKC levels and activity after ischemic injury, suggesting that PKC is degraded under these conditions^33^,^53^. The loss of total PKC activity, also seen in *in vitro* culture models of ischemic and excitotoxic cell death^54^,^55^, correlates with neurodegenerative processes^56^, implying that maintaining PKC activity may confer protection against excitotoxic damage. These apparently conflicting reports may stem from examination of varying animal models, brain regions, duration and intensities of the ischemia/reperfusion insult, and maybe compounded by the different, possibly opposing roles of individual PKC isozymes.

In the injured brain, activated microglia cells participate in the course of inflammation, a process that includes the actions of various kinds of cytokines. Some of these cytokines are necessary to protect neurons, others can be particularly harmful. Nonetheless, these actions depend on differences in polarization of microglia cells. Microglial cells, as the main immune cells of the CNS, are responsible for monitoring the brain microenvironment. Microglial activation results in the synthesis and secretion of a host of mediators, including prostaglandins (PGs), nitric oxide (NO) arising from upregulation of cyclo-oxygenase 2 (COX-2) and the inducible form of nitric oxide synthase (iNOS), respectively, as well as pro-inflammatory cytokines, such as interleukin-1 (IL-1), interleukin-6 (IL-6), and tumour necrosis factor alpha (TNF-α). This process is called persistent neuro-inflammation, or reactive gliosis, which develops in many acute and chronic neurological conditions, such as stroke, Parkinson’s and, Alzheimer’s disease, as well as motorneuron and prion diseases^57^,^58^,^59^. Excessive production of pro-inflammatory mediators such as cytokines, prostanoids, and free radicals, are thought to contribute to the neuropathological process and neuronal loss during ischemia. Inflammatory responses in brain ischemia/reperfusion lead to pivotal injuries in neurons that would eventually result in neuronal death. Mediators of inflammation released from microglial cells in the CNS are thus key mediators of ischemic brain injury.

IL-1β, IL-6 and TNFα are key pro-inflammatory cytokines that when induced can excessively activate microglial cells, forming a vicious cycle of pro-inflammatory responses that continuously damage neurons and other important nervous system structures. On the contrary, IL-4, IL-10, and TGF-β are important anti-inflammatory and pro-repare cytokines in the ischemic brain. Notably, these cytokines can preclude and reduce the imminent harm mediated by microglial cells after ischemic injury.

In this study, we investigated the immunity property of BV-2 microglial cells, and they match the immunity property of innate immune cells (Fig. S4) and found that pro-inflammatory cytokines are upregulated while anti-inflammatory cytokines are suppressed after phosphorylated ANXA1 is translocated to the nucleus of BV-2 microglial cells (Figs 6 and 7). This work highlights the importance of regulating pro-inflammatory cytokines and suggests a way in which we could preclude the translocation of ANXA1 into nucleus to protect neurons from death after ischemic injury. We are now evaluating possible therapeutic targets based on our results that can prevent neuronal cell loss in ischemic brain injury.

## Methods

### Cell culture

All animal experiments were approved by the Huazhong University of Science and Technology Institutional Animal Care and Use Committee, and performed in accordance with the National Institutes of Health Guide for the Care and Use of Laboratory Animals. Culture of primary microglial cells were performed as previously described^60^. Two freshly perfused adult mouse brains were used per experiment. Primary microglial cells were cultured in DMEM/F12 media with 10% FBS and 1% Pen/Step. The immortalized BV-2 microglial cell line was grown in Dulbecco’s Modified Eagle Medium (DMEM) with high glucose supplemented with 10% Fetal Bovine Serum (FBS), 100 U/ml penicillin and 100 μg/ml streptomycin at 37 °C in a 100% humidified atmosphere of 95% air and 5% CO_2_. Before all experimental procedures, BV-2 microglial cells were serum starved overnight (or for 12 h). Cells were then incubated with phorbol 12-myristate 13-acetate (PMA, 1 μM) (Beyotime, Shanghai, China) or GF103209X (GF, 1 μM) (PeproTech, Hamburg, Germany) for 30 min before cell protein was extracted.

### Oxygen glucose deprivation/re-oxygenation induced ischemic injury (OGD/R)

Culture medium was changed into glucose-free DMEM and washed with 1 X PBS three times, cultures were then transferred to an incubator containing 5% CO_2_ and 95% N_2_ at 37 °C for 1 h. Following Oxygen Glucose Deprivation (OGD) treatment, the cultures were re-oxygenated under normoxic conditions in high glucose-containing DMEM at37 °C in a humidified 5% CO_2_ incubator for 24 h before they were collected for analysis.

### Enzyme-Linked Immunosorbent Assay (ELISA)

Culture supernatants were collected after OGD/R treatments. The production of IL-1β, IL-6, IL-12, IL-17, TNF-α, IL-4, IL-10 and TGF-β were measured with a commercial ELISA kit (Biolegend, San Diego, CA) following manufacturers’ instructions and expressed as pg/mL.

### Immunocytochemistry

BV-2 microglia cells cultured on sterile glass cover slips were washed with 1 X PBS and fixed with 4% paraformaldehyde (PFA) for 15 min. The cells were then permeabilized with 0.2% Triton X-100 in 1 X PBS for 5 min. Cells were blocked in 1 X PBS containing 1% bovine serum albumin (BSA) for 30 min. The cells were incubated with primary antibodies overnight at 4 °C. The primary antibodies used were as follows: rabbit anti-ANXA1 (1:100, Santa Cruz Biotechnology, Dallas, TX), PKC (1:100, Santa Cruz Biotechnology, Dallas, TX). After washing, Alexa Fluor^®^ 594-conjugated anti- rabbit Ig G were applied at a dilution of 1:2000 for 1 h, and 4,6-diamidino-2-phenylindole (DAPI, Roche, Shanghai, China) was used for the identification of nuclei. Cover slips were mounted with glycerinum, and cells were imaged with an Olympus immunofluorescence microscope (Olympus, Tokyo, Japan).

### Immunoprecipitation and co-immunoprecipitation studies

Protein immunoprecipitation was performed according to manufacturer’s instructions of a commercially available immunoprecipitation kit, Protein A/G PLUS-Agarose, (Santa Cruz, Biotechnology, Dallas, TX) with only minor modifications. For this, cellular lysates were divided into two parts, one used for the immunoprecipitation assays and, the other for total protein analysis. For immunoprecipitation analysis, proteins were incubated with a polyclonal antibody against ANXA1 (1:200 (ug), Santa Cruz Biotechnology, Dallas, TX) at 4 °C on a vertical rotator overnight, followed by protein A/G plus agarose added (Santa Cruz, Biotechnology, Dallas, TX) for 4 h. After washing 5 times with lysis buffer, samples were eluted by boiling in 1 X SDS/PAGE buffer for 7 min. Proteins were then separated by SDS-PAGE and examined by immunoblotting using antibodies against PKC (1:1000, Santa Cruz Biotechnology, Dallas, TX), phosphor serine (1:250, Abcam, Cambridge, MA).

### Construction of expressive plasmids and ANXA1 site-directed mutagenesis

An ANXA1 cDNA construct containing a point mutation in a key phosphorylation site, 27 serine (S27) was generated. In addition, an ANXA1 cDNA construct tagged with a C-terminal enhanced green fluorescent protein (EGFP) was generated by attaching pEGFP-N1 [GenBank: U55762] to the wild-type (WT) construct between the restriction sites XhoI at the N-terminal coding region and the BamHI sites, replacing the stop codon. Replacement of the S27 amino acid with alanine (A) was introduced by sequential site-directed mutagenesis reactions employed the QuikChange site-directed mutagenesis kit (TransGen Bioteck, Beijing, China). Synthetic oligonucleotide primers containing the desired mutation site were extended in polymerase chain reaction (PCR) followed the kit protocol described. The products were DMT-treated to digest the parental template and transformed in DMT chemically competent *E. coli* cells. Plasmids were amplified in *E. coli* Trans5α cells (TransGen Bioteck, Beijing, China) and purified with a Plasmid Purification Kit (QIAGEN, Shanghai, China).

### Transfection of BV-2 microglial cells with ANXA1 plasmids

BV-2 microglia cells were plated in six-well plates in DMEM (Invitrogen, Carlsbad, CA) containing 10% FBS (Gibco) at 3 × 10^5^ cells per well for 24 h before transfection in a humidified atmosphere of 5% CO_2_ and 95% air at 37 °C. Cells were then transfected with ANXA1 cDNA using the expression plasmid, pEGFP-N1 (Invitrogen, Carlsbad, CA) according to the manufacturer’s introduction and, cells were harvested after a 48 h incubation period. All the cDNA plasmids described above were prepared using Endo-Free^®^ Plasmid Kits (QIAGEN, Shanghai, China) to avoid contamination of endotoxins.

### Cell surface protein preparation

For experimental assays in which cell surface ANXA1 was examined, we used previously described procedures^48^, which were modified briefly as follows. BV-2 microglial cells were first washed for 15 min on ice in HEPES buffer (25 mM) containing protease and phosphatase inhibitors (1 mM PMSF, 1 mg/ml leupeptin, 1 mg/ml pepstatin, 1 mg/ml aprotinin, 1 mM Na3VO4, 1 mM NaF; all from Sigma-Aldrich, St. Louis, MO), and a Ca^2^ chelating agent (1 mM EDTA-EGTA, Sigma-Aldrich, St. Louis, MO), which removes proteins attached to the cell surface. Subsequent washes were condensed with tubular ultrafiltration modules provided by Millipore (Billerica, MA), and retained for ANXA1 protein measurement.

### Western blot analysis

Cells were lysed in radioimmunoprecipitation assay (RIPA) buffer supplemented with a protease inhibitor cocktail (Roche, Shanghai, China) for whole cell protein preparations. Nuclear and cytoplasmic fractionations were performed with NE-PER Nuclear and Cytoplasmic Extraction Reagents (Thermo Scientific, Rockford, IL) according to the manufacturer’s protocol. Total protein concentration was measured and equal proteins were loaded and separated by 10% sodium dodecyl sulfate (SDS) polyacrylamide gel electrophoresis. Subsequently, proteins were transferred to PVDF membranes and then blocked with 5% milk or 5% BSA in Tris-buffered saline with Tween 20 (TBST) before immunodetection with the following antibodies: PKC (1:1000, Santa Cruz Biotechnology, Dallas TX), p-PKC (1:300, Cell Signaling Technology, Danvers, MA), ANXA1 (1:1000, Santa Cruz Biotechnology, Dallas TX), IL-1β (1:500, Abcam, Cambridge, MA), IL-6 (1:500, Abcam, Cambridge, MA), TNF-α(1:1000, Abcam, Cambridge, MA), IL-4 (1:500, Abcam, Cambridge, MA), IL-10 (1:500, Abcam, Cambridge, MA), TGF-β(1:1000, Abcam, Cambridge, MA), β-actin (1:4000, Santa Cruz Biotechnology, Dallas TX). After primary antibody incubation for 12 h, the PVDF membranes were washed with TBST (15–30 min at room temperature) before incubated with secondary antibody for 1 h. Specific binding was visualized by ECL reaction. The western blot bands were quantified using Image J Software (version 1.41).

### Statistical analysis

Data are expressed as means ± (SEM) of the indicated number of independent experiments. The statistical significance between multiple groups was analyzed by one-way ANOVA, the least significant difference (LSD) post hoc test was used for multiple comparisons, and the student’s t test was used to detect the significance of differences between two means. *P* < 0.05 was considered statistically significant.

## Additional Information

**How to cite this article**: Baoming, Z. *et al*. Annexin A1 translocates to nucleus and promotes the expression of pro-inflammatory cytokines in a PKC-dependent manner after OGD/R. *Sci. Rep.* **6**, 27028; doi: 10.1038/srep27028 (2016).

## Supplementary Material

Supplementary Information
