# Supplementary material for: Annexin A1 translocates to nucleus and promotes the expression of pro-inflammatory cytokines in a PKC-dependent manner after OGD/R
Source: Sci Rep. 2016 Jul 18;6:27028. doi: 10.1038/srep27028 (PMC4947919; doi:10.1038/srep27028)
Supplement: Supplementary Information [file srep27028-s1.pdf]

**Supplementary Information**

# Annexin A1 translocates to nucleus and promotes the expression of pro-inflammatory cytokines in a PKC-dependent manner after OGD/R

**Zhao Baoming, Jing Wang, Lu Liu, Xing Li, Shuangxi Liu, Qian Xia and Jing Shi***

## Methods

**Animals.** All animal experiments were approved by the Huazhong University of Science and Technology Institutional Animal Care and Use Committee, and performed in accordance with the National Institutes of Health Guide for the Care and Use of Laboratory Animals.

**Ischemia model**. Male C57BL/6 mice (Wuhan university laboratory animal center) were used for all experiments. Transient focal cerebral ischemia was induced by intraluminal occlusion of the left middle cerebral artery (MCA) for 60 minutes until reperfusion. In sham-operated animals, a silicon-coated nylon monofilament was withdrawn immediately after reaching the MCA to avoid ischemia. At 0.5 hours before MCAO, animals were randomly assigned to sham, GF109203X (GF, lateral ventricle injection of 10 μg/kg), and GF+MCAO groups.

**Flow cytometry.** Cells were treated with PE-Cy5-conjugated anti-CD11b, fluorescein isothiocyanate (FITC)-conjugated anti-MHC class I, FITC-conjugated anti-MHC class II, APC-conjugated anti-CD80 and PE-conjugated anti-CD86 antibodies or C57BL/6 mice control IgG (Biolegend, Copenhagen, Denmark) in PBS. We used standard gating strategies for fluorescence-activated cell sorting (FACS) using appropriate isotype control antibodies. Data was collected on a FACSCalibur™ (BD Biosciences) and analyzed using FACSDiva™ software version 6.1.2 (BD Biosciences).

## Results

**Brain ischemia/reperfusion induces serine phosphorylation of ANXA1.** Here, We detected serine phosphorylation levels of ANXA1 in brain tissue of a mouse model of middle cerebral artery occlusion (MCAO) by SDS-PAGE using a phospho-serine specific antibody. Serine phosphorylation levels of ANXA1 were significantly increased in the MCAO group when compared to sham treated animals ($n = 3$, $P < 0.05$). However, after injection of the PKC antagonist GF109203X (GF), serine phosphorylation levels of ANXA1 induced by MCAO were significantly reduced ($n = 3$, $P < 0.05$)。

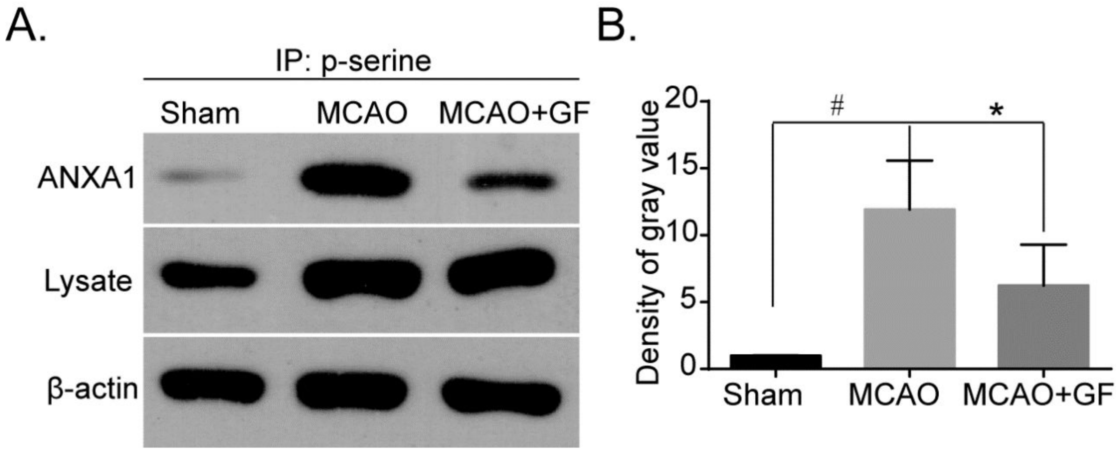


**Figure S1. Brain ischemia/reperfusion induces serine phosphorylation of ANXA1.** After MCAO, mouse brain tissue whole cell protein was extracted and analyzed by SDS-PAGE. Serine phosphorylation levels of ANXA1 in the MCAO group were markedly higher than those of the sham group, however, injection of PKC antagonist GF to inactivate PKC activity, significantly reduced serine phosphorylation levels of ANXA1 **(a)**. **(b)** Western blot intensities are quantified and normalized to their respective controls (defined as 1.0). Data are expressed as mean ± SEM; n = 3; #$P < 0.05$ vs. controls; *$P < 0.05$ vs. MCAO+GF groups.

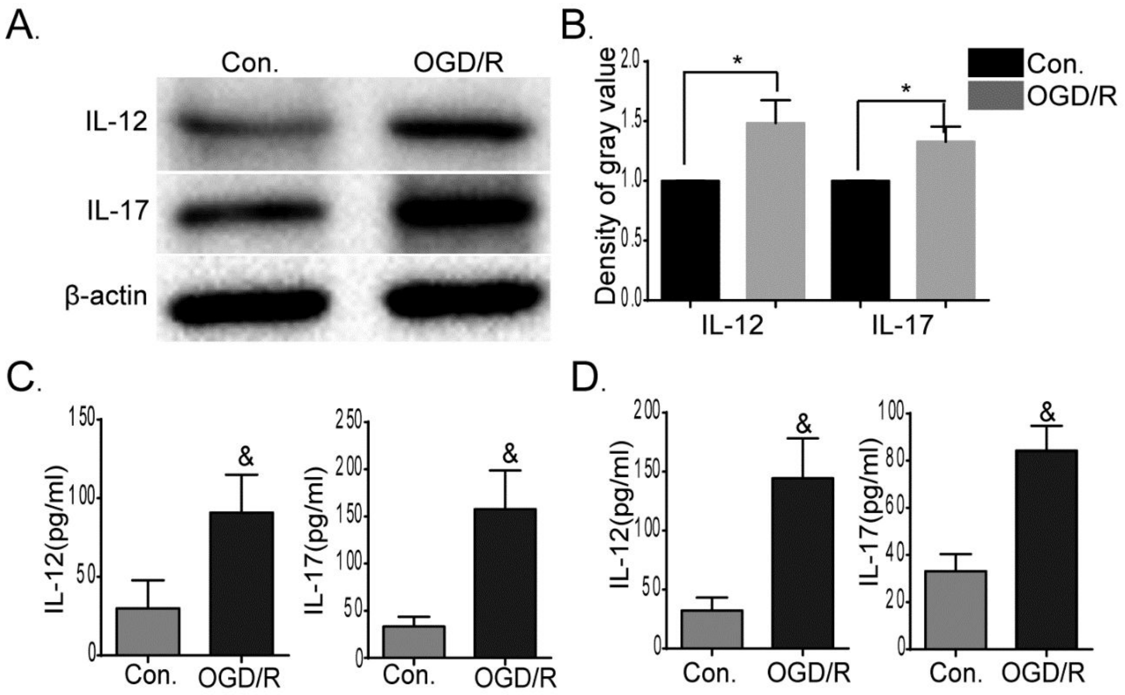


**Figure S2. Expression and secretion of IL-12/17 cytokines in BV-2 microglial cells and secretion of IL-12/17 cytokines in primary microglia treated by OGD/R.** We analyzed the expression levels of IL-12 and IL-17 in BV-2 microglial cells by SDS-PAGE, and their secretion by ELISA. **(a)** IL-12 and IL-17 expression levels in BV-2 microglial cells after OGD/R treatment were detected with anti-IL12 and anti-IL-17 monoclonal antibodies. **(b)** Quantification of western blot intensities normalized to their respective controls (defined as 1.0). Data are expressed as mean ± SEM; n = 3; *$P < 0.05$ vs. controls. **(c)** Data showing the secretion levels of IL-12 and IL-17 in the supernatants from BV-2 microglial and **(d)** primary microglial cells. Data are expressed as mean ± SEM; n = 3; &$P < 0.05$ vs. controls.

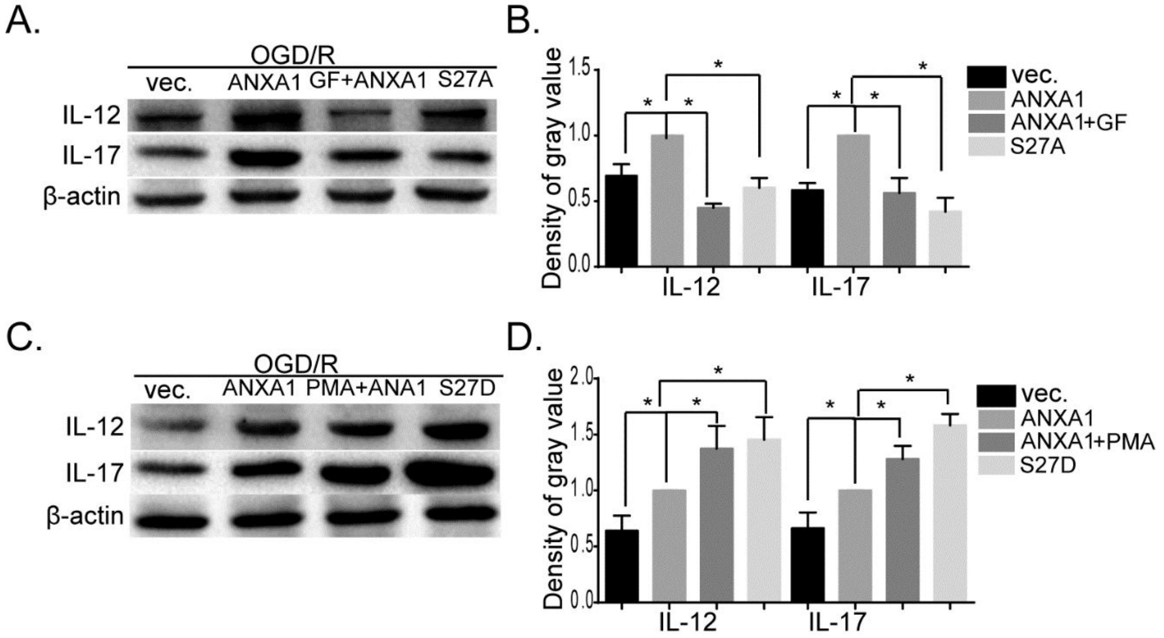


**Figure S3: Translocation of ANXA1 affects the expression of IL-12/17 cytokines in BV-2 microglia cells.** We used western blot analysis to examine the expression of IL-12 and IL-17 in BV-2 microglia cells. **(a)** BV-2 microglial cells were transfected with either vector control, WT ANXA1, or ANXA1-S27A mutant, or WT ANXA1 together with GF109203X (1 μM) after OGD/R treatments. **(b)** Western blot intensities of IL-12 and IL-17 were normalized to their respective controls (defined as 1.0). Data are presented as mean ± SEM for three independent experiments. Asterisks indicate statistically significant differences (*$P <$ 0.05). **(c)** BV-2 microglial cells were transfected with either vector control, or WT ANXA1, or with ANXA1-S27D, or WT ANXA1 and cells were treated with PMA (1 μM) after OGD/R treatments. **(d)** Western blot intensities of IL-12 and IL-17 normalized to their respective controls (defined as 1.0). Data are presented as mean ± SEM for three independent

experiments. Asterisks indicate statistically significant differences (*$P < 0.05$).

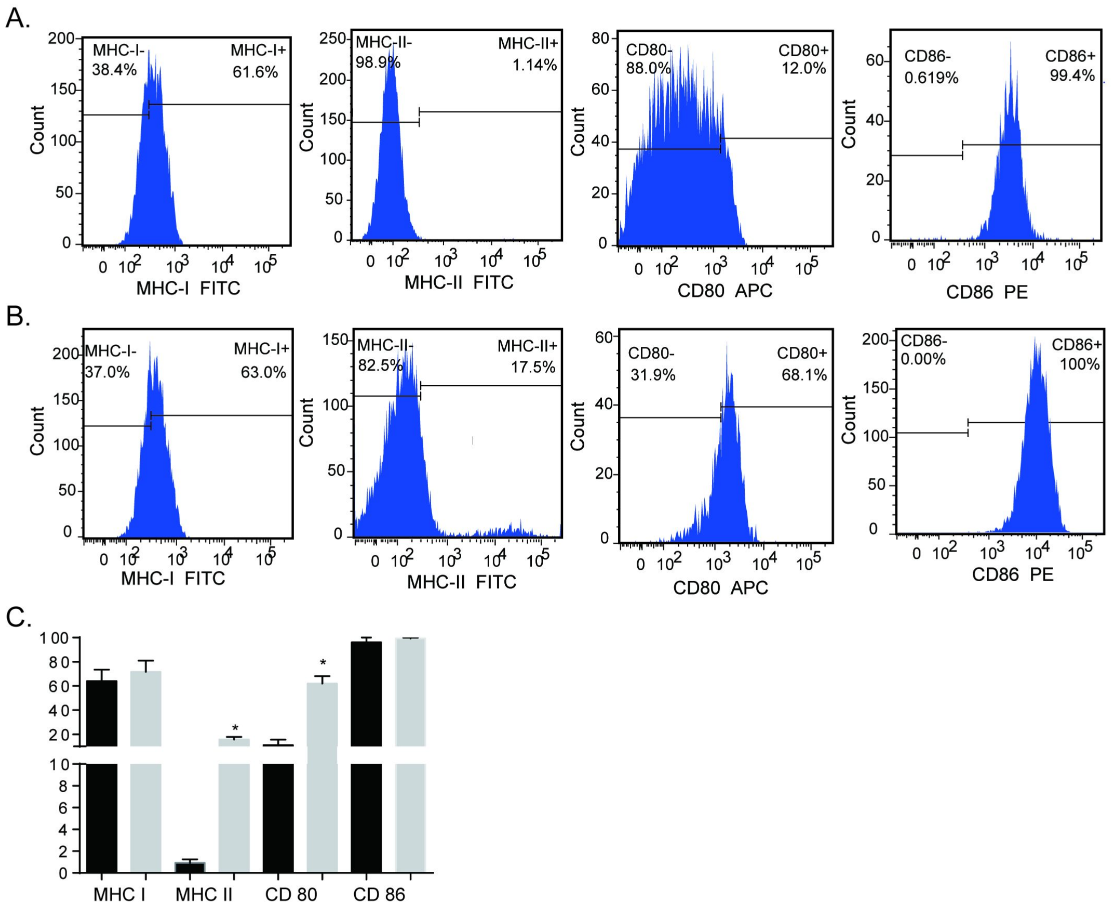


**Figure S4. Characterization of BV-2 microglial cells.** BV-2 microglial cells are stained for CD11b, major histocompatibility complex (MHC) class I, MHC class II, CD80 and CD86 and analyzed by flow cytometry in **(a)** Control and **(b)** OGD/R groups. **(c)** Data show that after OGD/R treatment, cell surface MHC class II and CD80 levels are significantly higher under these hypoxic conditions (*$P < 0.01$). In contrast, MHC class I levels do no significantly change under either hypoxic or

non-hypoxic conditions, whereas CD86 levels remain elevated throughout both conditions.
